# Synergistic Effects of Influenza and *Staphylococcus aureus* Toxins on Inflammation Activation and Cytotoxicity in Human Monocytic Cell Lines

**DOI:** 10.3390/toxins10070286

**Published:** 2018-07-11

**Authors:** Marion Jeannoel, Jean-Sebastien Casalegno, Michèle Ottmann, Cédric Badiou, Oana Dumitrescu, Bruno Lina, Gérard Lina

**Affiliations:** 1 Laboratoire de Virologie, Institut des Agents Infectieux, Groupement Hospitalier Nord des Hospices Civils de Lyon, CEDEX 04, 69317 Lyon, France; jean-sebastien.casalegno@chu-lyon.fr (J.-S.C.); bruno.lina@univ-lyon1.fr (B.L.); 2 Laboratoire de Virologie et Pathologies Humaines Virpath, CIRI, Centre International de Recherche en Infectiologie, Université de Lyon, Inserm U1111, CNRS UMR5308, École Normale Supérieure de Lyon, Université Claude Bernard Lyon 1, CEDEX 08, 69372 Lyon, France; michele.ottmann@univ-lyon1.fr; 3 Pathogénie des Staphylocoques, CIRI, Centre International de Recherche en Infectiologie, Université de Lyon, Inserm U1111, CNRS UMR5308, École Normale Supérieure de Lyon, Université Claude Bernard Lyon 1, CEDEX 08, 69372 Lyon, France; cedric.badiou@univ-lyon1.fr (C.B.); oana.dumitrescu-borne@univ-lyon1.fr (O.D.); 4 Laboratoire de Bactériologie, Institut des Agents Infectieux, Groupement Hospitalier Nord des Hospices Civils de Lyon, CEDEX 04, 69317 Lyon, France

**Keywords:** *Staphylococcus aureus*, toxins, influenza A virus, monocyte, coinfection, inflammation, cytotoxicity

## Abstract

In patients with influenza, morbidity and mortality are strongly influenced by infections with Staphylococcus aureus producing high amounts of certain toxins. Here we tested the impact of influenza virus on the pro-inflammatory and cytotoxic actions of a panel of S. aureus virulence factors, including Panton-Valentine Leucocidin (PVL), phenol-soluble modulin α1 (PSMα1) and 3 (PSMα3), α-hemolysin (Hla), and cell wall components, i.e., heat-killed S. aureus (HKSA) and protein A. We initially screened for potential synergic interactions using a standardized in vitro model in influenza-infected continuous human monocytic cell lines. Then we tested the identified associations using an ex vivo model in influenza-infected human monocytes freshly isolated from blood. Co-exposure to influenza virus and HKSA, PVL, PSMα1, and PSMα3 increased NF-κB/AP-1 pathway activation in THP1-XBlue cells, and co-exposure to influenza virus and PVL increased cytotoxicity in U937 cells. In monocytes isolated from blood, the synergy between influenza virus and HKSA was confirmed based on cytokine production (TNF-α, IL-1β, IL-6), and co-exposure to influenza virus and Hla-increased cytotoxicity. Our findings suggest that influenza virus potentiates the pro-inflammatory action of HKSA and contributes to the cytotoxicity of Hla on monocytes. Synergic interactions identified in the cell-line model must be cautiously interpreted since few were relevant in the ex vivo model.

## 1. Introduction

The World Health Organization estimates that influenza is responsible for 3 to 5 million cases of severe illness and 250,000 to 500,000 deaths per year worldwide [1]. Influenza-related morbidity and mortality are mainly associated with secondary bacterial infections [2,3]. *Staphylococcus aureus* has recently emerged as a major pathogen in influenza virus superinfection [4,5], seemingly concomitant with the emergence of community-acquired methicillin-resistant *S. aureus* (CA-MRSA) since the early 2000s [6].

CA-MRSA strains produce a diverse arsenal of virulence factors that contribute to the pathogenesis of *S. aureus* lung infection. The pathogen-associated molecular patterns (PAMPs) are recognized by Toll-like receptors (TLR) and other pattern recognition receptors, prompting activation of innate immune responses [7]. Virulence determinants involved in the pathophysiology of *S. aureus* lung infection include *S. aureus* PAMPs, such as cell-wall anchored lipoproteins, lipoteichoic acid, peptidoglycan, and protein A; and excreted toxins, such as alpha-toxin (Hla), Panton-Valentine Leukocidin (PVL), and α-type phenol-soluble modulins (PSMα). These factors activate the immune system through different receptors, but all trigger the NF-κB pathway and release of pro-inflammatory mediators [8,9,10,11,12,13]. Recognition of influenza virus nucleic acids by TLR3, 7, and 8 also leads to NF-κB pathway activation [14,15].

We do not yet fully understand the pathogenic mechanisms through which influenza virus infection increases both host susceptibility and severity of *S. aureus* super-infection. Experimental in vivo models of post-influenza pneumonia suggest that most respiratory tract lesions are induced by an enhanced inflammatory response from immune cells recruited in the lungs, and their subsequent destruction [16,17,18]. The initial immune response is characterized by monocyte/macrophage recruitment into the lung parenchyma and alveolar spaces, which is necessary for host protection and recovery. However, excessive recruitment of these cells may contribute to potentially lethal lung pathology [19,20,21]. In severe infection, severe lung damage is accentuated by early and excessive production of type I interferons, amplifying the MCP-1 production responsible for inflammatory monocyte recruitment [22]. In human peripheral blood mononuclear cells (PBMCs) exposed to influenza virus, type I interferons also increase the expression of functional tumor necrosis factor-related apoptosis-inducing ligand (TRAIL), thereby increasing the sensitivity to TRAIL-induced apoptosis in influenza-infected cells [23]. The inflammatory response mediated by increased monocyte recruitment to the lung is the main determinant of lung damage, more so than influenza virus replication [23,24].

Little information is presently available regarding the interactions between *S. aureus* toxins and the influenza virus at the cellular level. Therefore, in this study, we aimed to evaluate the potential synergic effects of influenza virus and *S. aureus* virulence factors on inflammation and cytotoxicity against human monocytes. We initially screened the potential synergic interactions using a standardized model of influenza-infected continuous human monocytes. Then we tested the significant associations using a more relevant model of influenza-infected primary human monocytes.

## 2. Results

### 2.1. Co-Exposure of THP1-XBlue Cells to Influenza Virus S. aureus Virulence Determinants (PVL, PSMα1, PSMα3, Protein A, and HKSA) Is Associated with Higher NF-κB/AP-1 Pathway Activation than Exposure to Influenza Alone

**Figure 1:**
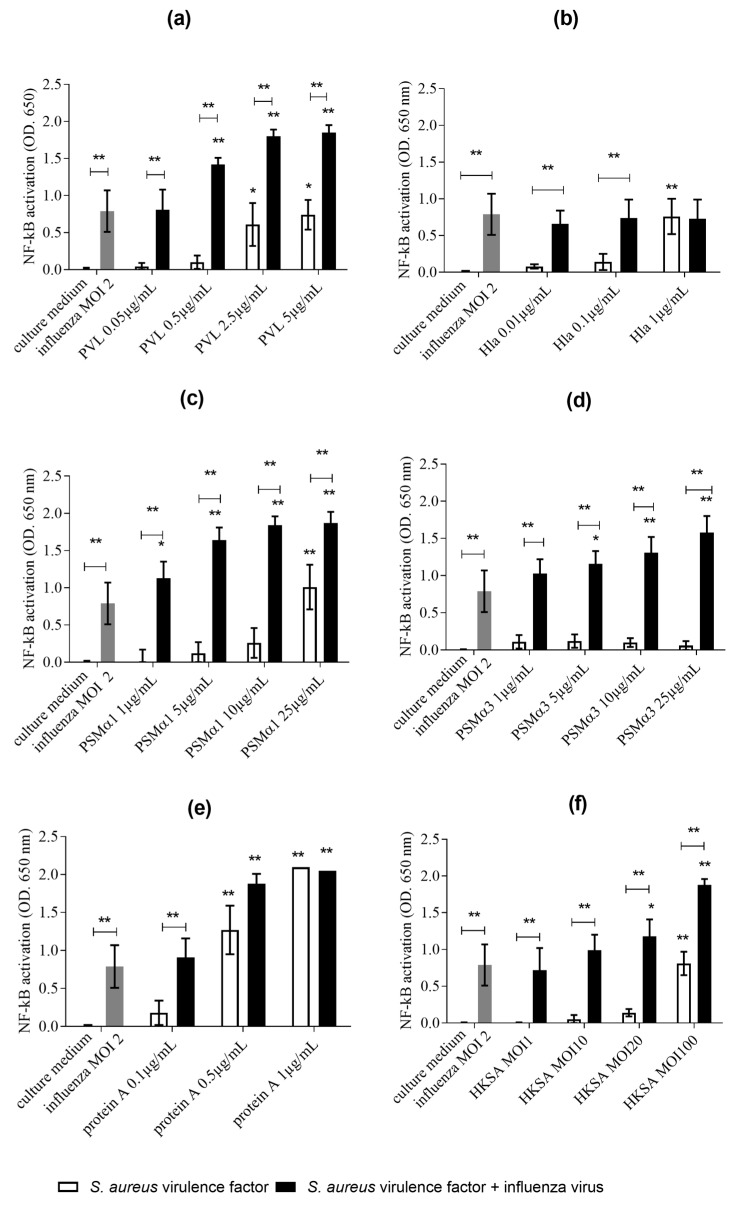
Activation of NF-κB/AP-1 in THP1-XBlue cells by influenza virus and/or *S. aureus* virulence factors measured after a 24-h incubation period. THP1-XBlue cells were treated with influenza virus (MOI 2), and (**a**) PVL (0.05, 0.5, 2.5, and 5 µg/mL), (**b**) Hla (0.01, 0.1, and 1 µg/mL), (**c**) PSMα1 (1, 5, 10, and 25 µg/mL), (**d**) PSMα3 (1, 5, 10, and 25 µg/mL), (**e**) protein A (0.1, 0.5, and 1 µg/mL), and (**f**) HKSA (MOI 1, 10, 20, and 100). SEAP activity was measured as a proxy for NF-κB/AP-1 activation. Data are representative of three independent experiments and each condition was tested in duplicate. Error bars represent SD (*n* = 6). ANOVA and the Bonferroni post-hoc test were used to verify statistical significance. * *p* < 0.05, ** *p* < 0.01. Each toxin concentration was compared to the culture medium, and each toxin concentration + influenza virus was compared to influenza virus alone, as indicated by stars. Each toxin concentration was compared to the same concentration of toxin + influenza virus as indicated by lines. Each concentration of toxin was compared to the culture medium, each concentration of toxin + influenza virus was compared to influenza virus alone as indicated by stars. Each concentration of toxin was compared to the same concentration of toxin + influenza virus as indicated by lines. OD 650, optical density at 650 nm.

We first incubated influenza virus-exposed and non-exposed THP1-XBlue cells for 24 h with sublytic concentrations of *S. aureus* products (PVL, protein A, HKSA, Hla, PSMα1, and PSMα3), and compared the NF-κB/AP-1 pathway activation. Compared to the cells exposed only to *S. aureus* virulence factors, the THP1-XBlue cells co-exposed to influenza virus and the tested virulence factors (except Hla) showed increased NF-κB/AP-1 activation by 2- to 10-fold (Figure 1). In influenza-exposed cells, the lowest concentrations of toxins that triggered significant NF-κB/AP-1 activation were PVL 0.5 µg/mL (vs. 2.5 µg/mL in non-influenza-exposed cells), PSMα1 1 µg/mL (vs. 25 µg/mL), and PSMα3 5 µg/mL (vs. no activation) (Figure 1). Co-exposure of the cells to influenza virus at a multiplicity of infection (MOI) of 2, and to PVL (2.5 µg/mL), HKSA (MOI 100), and PSMα1 (10 µg/mL) yielded NF-κB/AP-1 activation to the same extent as that induced by the most potent activator (protein A, 1 µg/mL). Although influenza virus alone and *S. aureus* virulence factors alone had only a modest effect (OD < 1) on NF-κB/AP-1 activation, our findings suggested that co-exposure of a continuous monocytic cell line to both influenza virus and *S. aureus* virulence factors potentiated the pro-inflammatory activity.

### 2.2. In Monocytes Isolated from Human Peripheral Blood, Co-Exposure to Influenza Virus and HKSA Is Associated with Enhanced Cytokine Production (TNF-α, IL-1β, and IL-6)

**Figure 2:**
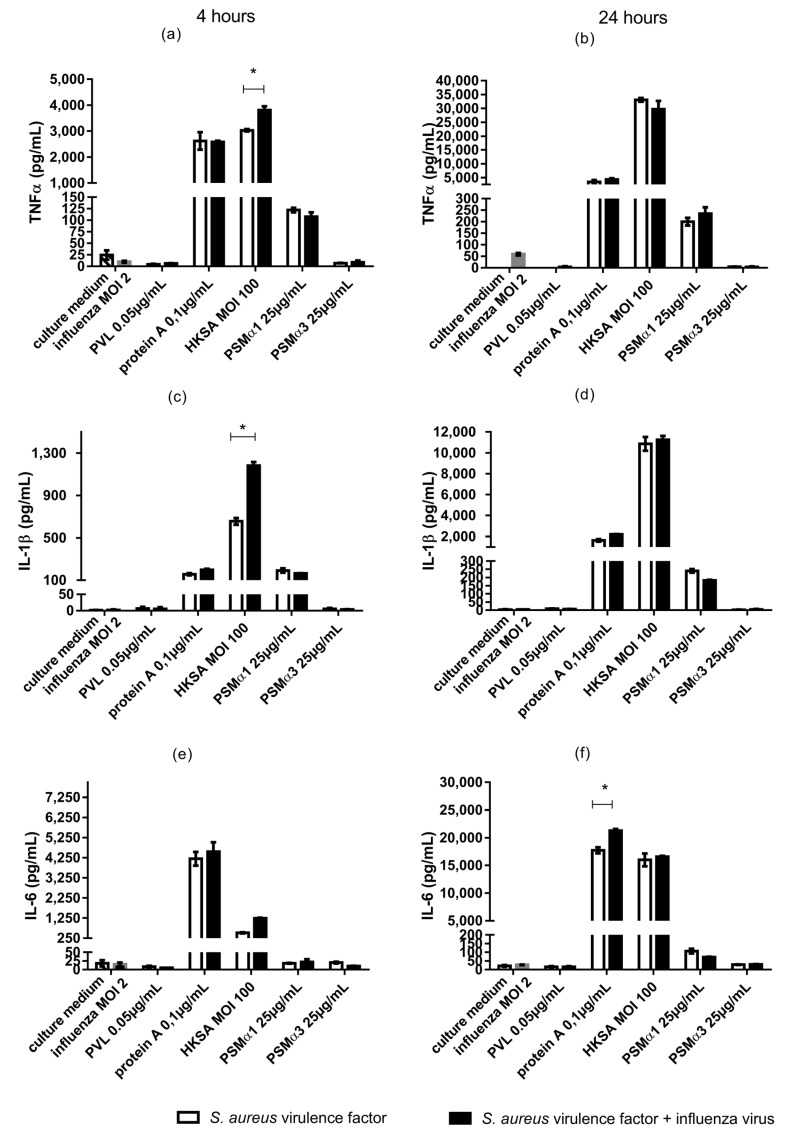
Cytokine concentrations measured from supernatants of monocytes isolated from human peripheral blood, co-exposed to influenza virus and *S. aureus* virulence determinants (PVL, Hla, SpA, HKSA, PSMα1, and PSMα3). (**a**) TNF-α after a 4-h incubation. (**b**) TNF-α after a 24-h incubation. (**c**) IL-1β after a 4-h incubation. (**d**) IL-1β after a 24-h incubation. (**e**) IL-6 after a 4-h incubation. (**f**) IL-1β after a 24-h incubation. Monocytes isolated from human peripheral blood were treated with influenza virus (MOI 2), PVL (0.05 µg/mL), protein A (0.1 µg/mL), HKSA (MOI 100), PSMα1 (25 µg/mL), or PSMα3 (25 µg/mL). Data are representative of three independent experiments. Error bars represent SD (*n* = 3). ANOVA and the Bonferroni post-hoc test were used to verify statistical significance. * *p* < 0.05. Cytokine concentrations determined for supernatants exposed to one *S. aureus* virulence determinant condition were compared to the culture medium. Cytokine concentrations determined for supernatants exposed to one *S. aureus* virulence determinant + influenza virus were compared to influenza virus alone, as indicated by stars. Cytokine concentrations determined for supernatants exposed to one *S. aureus* virulence determinant condition were compared to supernatants co-exposed to influenza virus, as indicated by lines. OD 450, optical density at 450 nm.

**Figure 3:**
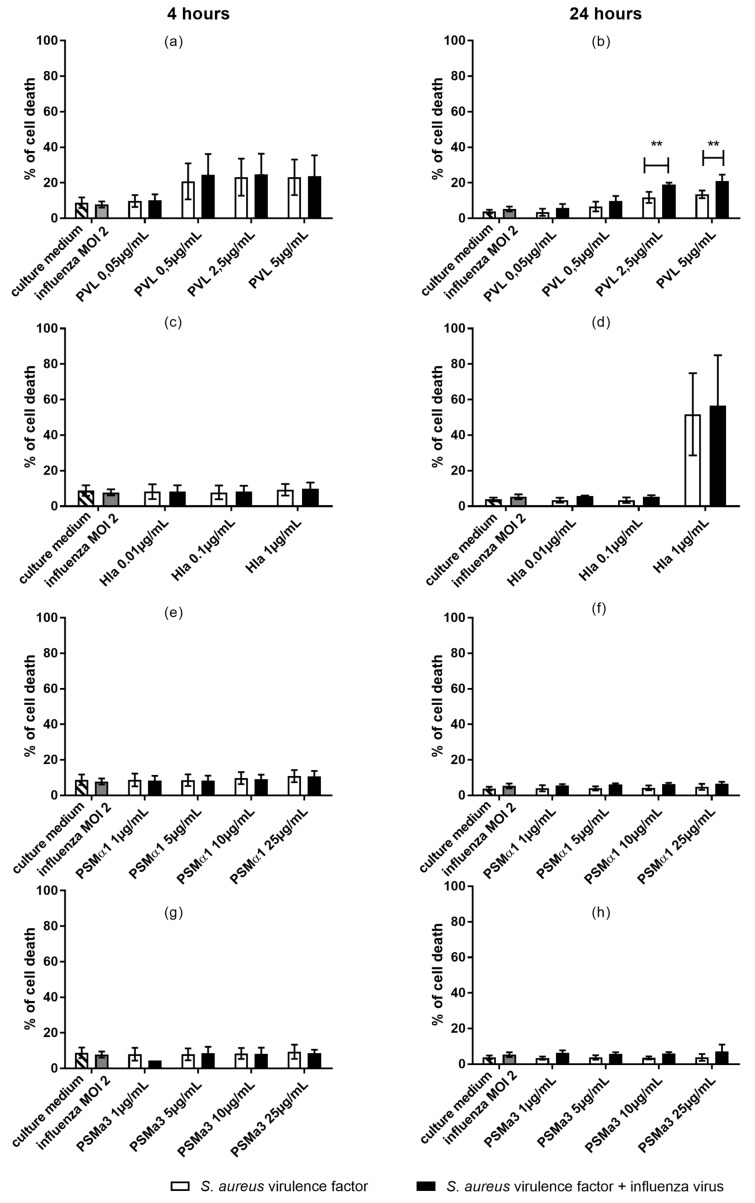
Cytolytic effects of influenza virus and *S. aureus* toxins on U937 cells after 4- and 24-h incubation periods. The monocyte population was gated using forward and side scatters (FSC and SSC, respectively). Cells were exposed to influenza virus (MOI 2) and (**a**,**b**) PVL (0.05 to 5 µg/mL), (**c**,**d**) Hla (0.01 to 1 µg/mL), (**e**,**f**) PSMα1 (1 to 25 µg/mL), and (**g**,**h**) PSMα3 (1 to 25 µg/mL). Propidium iodide (PI) incorporation was used to measure the % cell death within the gated monocyte population. Data are representative of three independent experiments, and each condition was tested in duplicate. Error bars represent SD (*n* = 6). Each toxin concentration was compared to culture medium, and each toxin concentration + influenza was compared to influenza virus alone, as indicated by stars. Each toxin concentration was compared to toxin + influenza virus as indicated by lines. ANOVA and the Bonferroni post-hoc test were used to verify statistical significance. ** *p* < 0.01.

**Figure 4:**
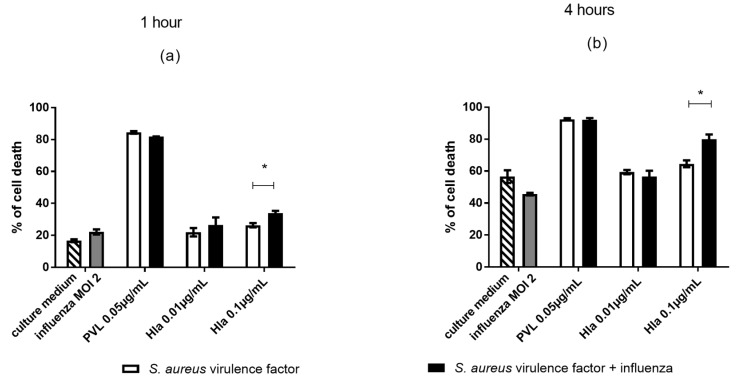
Cytolytic effects of influenza virus and *S. aureus* toxins on human monocytes after 1- and 4-h incubation periods. The monocyte population was gated using forward and side scatters (FSC and SSC, respectively). Cells were exposed to influenza virus (MOI 2) and PVL (0.05 µg/mL) or Hla (0.01 to 0.1 µg/mL) for (**a**) 1 h or (**b**) 4 h. Propidium iodide (PI) incorporation was used to measure the % cell death within the gated monocyte population. Data are representative of three independent experiments, and each condition was tested in duplicate. Error bars represent SD (*n* = 6). Each toxin concentration was compared to culture medium, and each toxin concentration + influenza virus was compared to influenza virus alone, as indicated by stars. Each toxin concentration was compared to toxin + influenza virus, as indicated by lines. ANOVA and the Bonferroni post-hoc test were used to verify statistical significance. * *p* < 0.05.

The pro-inflammatory synergistic associations identified in continuous cell lines were next tested in a model of human primary monocytes. Monocytes were isolated from human peripheral blood, co-exposed to influenza virus and *S. aureus* virulence determinants (PVL, protein A, HKSA, PSMα1, and PSMα3), and we measured production of the cytokines TNF-α, IL-1β, and IL-6 in the supernatants after 4- and 24-h incubation periods. We tested the combinations that showed the most potent synergic effects in continuous cell lines: influenza virus (MOI 2) plus PSM α1 (25 µg/mL), PSM α3 (25 µg/mL), and HKSA (MOI 100) (Figure 2). Since protein A is a strong inflammation inducer, we used the lowest protein A concentration that showed synergy with influenza virus on inflammation (0.1 µg/mL). We used the PVL concentration having the lowest cytolytic action on human monocytes (0.05 µg/mL) (Figure 3 and Figure 4).

Protein A, HKSA, and PSMα1 induced significant cytokine production from human monocytes (Figure 2). Under the tested conditions, protein A and HKSA were potent inducers of TNFα, IL-1β, and IL-6 production from monocytes as early as 4 h after exposure to toxins. Exposure to influenza virus alone did not induce significant cytokine production. The cytokine levels in human peripheral blood supernatant did not significantly differ following exposure to PVL, PSMα1, and PSMα3 alone versus co-exposure to these toxins and influenza virus (Figure 2).

In monocytes exposed to HKSA for 4 h, co-exposure to influenza virus significantly increased TNFα and IL-1β production. Similarly, in monocytes exposed to protein A for 24 h, co-exposure to influenza virus significantly increased IL-6 production (Figure 2). These results suggest that influenza virus may potentiate HKSA and protein A pro-inflammatory activity in human monocytes in vivo. Unlike in THP1-XBlue cells, co-exposure to influenza virus did not significantly increase cytokine production in human monocytes exposed to PVL, PSMα1, PSMα3.

### 2.3. In U937 Cells, Co-Exposure to Influenza and PVL is Associated with Increased Cytotoxicity

We next co-stimulated THP1 monocytes with influenza virus (MOI 2) and sublytic to lytic concentrations of cytolysins (PVL, Hla, PSMα1, and PSMα3), with the aim of testing whether co-exposure to influenza virus could influence the cytotoxicity of *S. aureus* toxins towards monocytes. After 4- and 24-h incubation periods, we examined the toxicity of these conditions by staining THP1 cells with PI and using flow cytometry to detect necrosis-like membrane damage on monocytes. THP1 cells were found to be highly resistant to *S. aureus* toxins (only 7% cell death observed using the higher toxin concentrations; data not shown); therefore, we switched to U937 cells (Figure 3).

Influenza virus alone did not significantly enhance cell death relative to the culture medium alone. We observed cytotoxicity following exposure to lytic concentrations of PVL (2.5 and 5 µg/mL) and Hla (1 µg/mL), and residual cytotoxicity following exposure to the tested concentrations of PSMα1 and PSMα3 (Figure 3). Under these conditions, Hla was the most potent cytolysin, inducing 52% cell death. In contrast, PVL induced only 14% cell death (Figure 3). Co-exposure of monocytes to influenza virus and PVL (2.5 and 5 µg/mL) during a 24-h incubation resulted in significantly increased cell death compared to the activity of PVL alone (20% vs. 14% and 25% vs. 15%). These results suggested that influenza virus infection of monocytes increased the cells’ susceptibility to PVL-induced cytotoxicity. Cell death was not increased by co-exposure of cells to influenza virus and the other toxins (Hla, PSMα1, and PSMα3).

### 2.4. In Monocytes Isolated from Human Peripheral Blood, Co-Exposure to Influenza Virus and Hla Is Associated with Increased Cytotoxicity

The cytolytic associations between influenza virus and PVL or Hla that were identified in continuous cell lines, were subsequently tested in a model of human primary monocytes. Compared to U937 cells, primary monocytes are much more sensitive to cytolysins; therefore, we selected low concentrations of PVL (0.05 µg/mL) and Hla (0.01 and 0.1 µg/mL). After 1- and 4-h incubation periods, monocytes were stained with PI, and toxicity was assessed by flow cytometry. Under our test conditions, we observed maximal cytotoxicity after the 4-h incubation period and thus did not test after a 24-h incubation period. PVL showed the most potent cytolytic activity, inducing 80% cell death at the lowest tested concentration (0.05 µg/mL) after as little as 1 h of PVL exposure (Figure 4). Hla (0.1 µg/mL) induced significant cell death at both 1 h and 4 h. In contrast, influenza virus infection of monocytes did not induce significant cell death.

Co-exposure of cells to influenza virus and a lytic concentration of Hla induced a significant increase in cell death. These findings suggested that influenza viral infection of monocytes increased the susceptibility of these cells to Hla cytotoxicity. Under our experimental conditions, PVL cytotoxicity was already too effective to detect any additional synergic effect of influenza virus on monocyte sensitivity to PVL. These results were confirmed by measurement of LDH release in monocytes supernatants, as we found no significant difference between monocytes exposed to cytolysin alone or co-exposed to cytolysin and influenza virus (data not shown).

**Figure 5:**
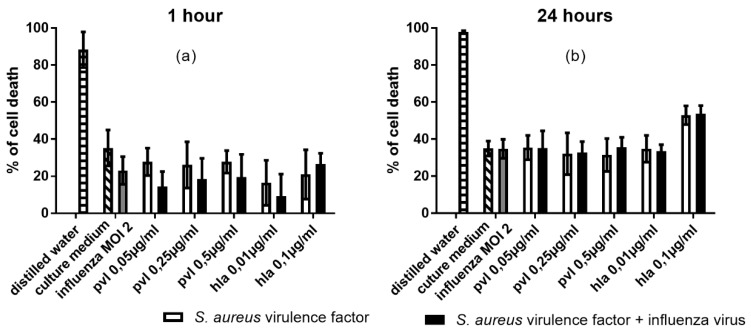
Effect of supernatants from monocytes exposed to influenza virus and *S. aureus* toxins on A549 cells after incubation periods of (**a**) 1 h and (**b**) 24 h. Supernatants of monocytes after 24-h exposure to influenza virus (MOI 2), PVL (0.05, 0.25, and 0.5 µg/mL), and Hla (0.01 and 0.1 µg/mL) were used to incubate epithelial cell monolayers for 24 h. Then the percentage of viable cells was estimated based on neutral red uptake assay. Data are representative of three independent experiments, and each condition was tested in duplicate. Error bars represent SD (*n* = 6).

Next, we tested how the supernatant of monocytes exposed to influenza virus and *S. aureus* cytolytic toxins affected confluent monolayers of A549 cells after incubation for 1 h and 24 h. After a 1-h incubation period, monocyte supernatant did not affect epithelial cell viability compared to control. After a 24-h incubation period, Hla showed a tendency to increase A549 mortality, but this trend did not achieve statistical significance (Figure 5).

## 3. Discussion

Broad pulmonary inflammatory infiltration is a key clinical feature of bacterial pneumonia following influenza viral infection. Lung histopathological analyses in murine models reveal severe lung injuries in cases of co-infections [16,17]. The influenza virus and *S. aureus* virulence factors induce inflammation via different cellular pathways that all converge to activation of the NF-κB classical pathway [8,9,10,11,12,14,15,16,25] and the subsequent secretion of inflammatory cytokines [26,27]. Most related studies have been conducted using animal models, and despite the convergence to the NF-κB pathway, few studies have examined the impact of various *S. aureus* toxins on human leukocytes exposed to influenza virus [28].

In our present study, we tested a broad range of *S. aureus* virulence factors—including PVL, Hla, PSMs, protein A, and cell wall components which are involved in the pathophysiology of *S. aureus* lung infection [29,30]. We performed our initial screening in a standardized in vitro model of influenza virus-infected continuous human monocytic cell lines, and subsequent testing in an ex vivo model of influenza virus-infected human monocytes. Our results in infected human primary monocytes demonstrated a new synergic action between influenza virus and HKSA on the induced inflammation, as well as synergy between influenza virus and Hla on the induced cytotoxicity.

Our results also highlighted the importance of validating results obtained in continuous monocytic cell lines in a more relevant ex vivo model of influenza virus-infected human monocytes. Indeed, cell susceptibility seemed to differ between continuous cell lines and human monocytes freshly isolated from blood. We observed synergy between influenza virus and all the tested toxins (except Hla) in THP1-XBlue cells. On the other hand, under similar conditions, influenza virus showed synergy with only HKSA and protein A in freshly isolated monocytes as determined based on increased inflammation through cytokine production. Moreover, cytotoxicity induced by PVL and Hla differed between U937 cells, THP1 cells, and blood-isolated monocytes. While influenza virus and PVL showed additive effects on cytotoxicity in U937 cells, this effect could not be detected in monocytes due to their extreme sensitivity to PVL alone, whereas additive cytotoxicity was observed between Hla and influenza virus in monocytes. These differences demonstrate the importance of the choice of a cellular model for these experiments.

The PVL and Hla concentrations used in our study are similar to those reported in vivo [29,31]. The literature does not include any reported in vivo concentrations of protein A, PSMα1, or PSMα3. We used concentrations of protein A and influenza virus that are similar to those currently employed in vitro to induce inflammation in monocytic cell lines [9,22,31]. We observed that PSMα1 and PSMα3 concentrations of above 10 µg/mL were required for NF-κB pathway activation, which is higher than the concentrations measured in vitro in culture supernatants of various *S. aureus* strains [32]. To our knowledge, *S. aureus* PSMs have been demonstrated to have inflammatory properties on polymorphonuclear cells but not monocytes [32,33].

Clinical data support that PVL and Hla may play a major role in severe pediatric MRSA pneumonia associated with respiratory viral infection [34,35]. These two pore-forming toxins have inflammatory actions through osmotic stress that leads to NLRP3 activation and consequently to IL-1β production [24,36]. Here we observed that PVL induced a proinflammatory response in THP1-XBlue cells. However, unlike in the literature, we did not observe IL-1β production in supernatants of human monocytes [24,37]. This difference could be explained by the fast lysis of monocytes after their exposure to the toxin. Indeed, pro-IL-1β is not constitutively expressed in monocytes but must be produced following the activation of pattern recognition receptors by microbial products. After NLRP3 activation, pro-IL-1β is cleaved into mature and active IL-1β [38]. Thus, early cytotoxicity could prevent cells from producing IL-1β. We observed that HKSA induced robust IL-1β production from monocytes, and that exposure to HKSA with influenza virus co-infection increased the early IL-1β production of monocytes. This would contribute to disease severity, since higher IL-1β levels have been associated with poor outcomes in acute respiratory distress syndrome [39]. The capacity of HKSA to induce IL-1β production by monocytic cells seems to be linked to the structure of *S. aureus* PGN and its degradability by immune cells. This could explain the discrepancies reported in the literature [24,40,41]. 

To investigate cytotoxicity, we had to use U937 cells because THP1 cells were highly resistant. In this model, we observed that high toxin concentrations were required to observe their cytolytic properties: 2.5 µg/mL PVL and 1 µg/mL Hla. We observed no cytotoxicity with PSMs, which are only weakly cytolytic, with the exception of PSMα3 [32]. These results were not unexpected, since U937 cells are relatively resistant to lysis compared to primary blood-derived monocytes. [24]. We further observed that pre-exposure to influenza virus resulted in an almost 2-fold increase in the PVL-related death of U937 cells. In contrast, we did not observe any additive effect in cells co-exposed to influenza virus and to the other tested toxins (Hla, PSMα1, and PSMα3). It appears that the contribution of PSM to post-influenza pneumonia is likely linked to epithelial cell damage rather than monocyte damage [30]. Under our experimental conditions, human monocytes were already highly sensitive to the utilized PVL concentrations, preventing us from observing any potential synergy between influenza virus and PVL on primary monocytes. However, we observed synergy between Hla and influenza virus infection. It can be assumed that monocyte death results in a release of proteases that contribute to lung lesions, as has been shown for polymorphonuclear cells [28].

The synergy observed between influenza virus and HKSA or protein A regarding cytokine production presumably promotes increased leukocyte recruitment to the lungs. Subsequent large-scale lysis of these recruited immune cells by *S. aureus* cytolysins can thus be expected. The presently available data warrant further investigation of the roles of monocytes in the inflammation observed during coinfection. To date, monocytes have been less studied in this context compared to polymorphonuclear cells.

The influenza virus may potentiate the actions of *S. aureus* toxins and PAMPs actions in two ways. First, the influenza virus increases the inflammatory potential of some pro-inflammatory toxins. Thus, monocytes reinforce chemokine production, leading to recruitment of neutrophils and monocytes, and massive migration of macrophages to the lung [26,27]. Second, the virus increases the cytotoxic potential against these recruited cells. Influenza virus and *S. aureus* toxins have a synergistic pro-inflammatory impact on human monocytes and are linked to increased Hla cytotoxicity against influenza virus-exposed human monocytes. This combination of excessive inflammation and increased cytotoxicity contributes to more severe outcomes. Thus, severe pneumonia with worse disease outcomes is associated with CA-MRSA, such as the USA300 strain, which presents increased PSM and Hla production and, more frequently, PVL production compared to methicillin-sensitive *S. aureus* strains (MSSA) [32,42,43,44]. Excessive inflammatory responses after establishment of secondary bacterial infection represent another difficulty in the clinical management of disease, and is a likely reason for enhanced disease severity and mortality despite appropriate antibiotic treatment [45,46]. This suggests that immunomodulators or neutralizing antibodies may useful for controlling excessive inflammation and improving clinical outcome.

## 4. Materials and Methods

### 4.1. S. aureus Virulence Factors

To obtain heat-killed *S. aureus* (HKSA), the LUG 960 strain (RN6390 delta *spa::kan*) was obtained from the French national reference center for staphylococci (*Centre national de référence des Staphylocoques*, Lyon, France) and heated to 100 °C for 1 h. Recombinant PVL was produced and purified by the *Centre national de référence des Staphylocoques*. Hla and protein A were obtained from Sigma-Aldrich (L’Isle d’Abeau, France). PSMα1 and PSMα3 were synthesized by GeneCust (Dudelange, Luxembourg). We tested sublytic to lytic concentrations of the cytotoxins PVL, Hla, PSMα1, and PSMα3, as described in the literature [24,31,34]. The utilized concentrations of protein A, HKSA, and influenza virus were similar to those currently employed in vitro to induce inflammation in monocyte cell lines [9,22,31].

### 4.2. Influenza Virus

The human influenza virus A/Puerto Rico/8/34 (A/PR/8/34; H1N1; ATCC VR-1469) was propagated and titrated in Madin-Darby Canine Kidney cells (MDCK; ATCC, CCL34) as previously described [47]. Briefly, MDCK cells were maintained at 37 °C under 5% CO_2_, in Ultra-MDCK serum-free medium (Lonza, Levallois, France) supplemented with 2 mM l-glutamine, 200 IU/mL penicillin (Lonza, Levallois, France), and 200 IU/mL streptomycin (Lonza, Levallois, France). The A/PR/8/34 strain was propagated in MDCK cells at 34 °C in EMEM (Lonza, Levallois, France) supplemented with 1 µg/mL trypsin (Sigma-Aldrich, St. Quentin Fallavier, France). The supernatant was harvested, and the virus titer was assessed by endpoint titration in MDCK cells.

### 4.3. Culture of THP1, THP1-XBlue, and U937 Cells

THP1 cells (ATCC^®^ TIB-202^TM^) were grown at 37 °C and 5% CO_2_ in RPMI 1640 medium (Eurobio, Courtaboeuf, France) supplemented with 10% fetal bovine serum (FBS, Gibco Life Technologies, Saint Aubin, France) and 50 μg/mL Pen-Strep (Invivogen, Toulouse, France). THP1-XBlue cells, which contain a secreted embryonic alkaline phosphatase (SEAP) reporter gene under the control of NF-κB and AP-1, were acquired from Invivogen and grown at 37 °C and 5% CO_2_ in RPMI 1640 medium supplemented with 10% FBS, 100 µg/mL Normocin^TM^, and 50 μg/mL Pen-Strep. THP1-XBlue. U937 cells (ATCC^®^ CRL-1593.2^TM^) were grown at 37 °C and 5% CO_2_ in RPMI 1640 medium supplemented with 10% FBS and 50 μg/mL Pen-Strep.

### 4.4. Monocyte Isolation from Human Peripheral Blood

Blood samples were collected from healthy donors who gave their informed consent, with ethics committee approval (Etablissement Français du Sang, Lyon, France). Human monocytes were isolated with a purity of >95% using RosetteSep human monocyte enrichment cocktail (Stemcell technologies, Grenoble, France) following the manufacturer’s protocol. Isolated monocytes were resuspended at a final concentration of 0.5 × 10^6^ cells/mL in RPMI 1640 medium supplemented with 10% FBS and 50 μg/mL Pen-Strep.

### 4.5. Stimulation of Cell Lines

THP1-XBlue, U937, or THP1 cells were plated in 96-well plates (2 × 10^5^ cells per well) in RPMI 1640 with 10% FBS. We used heat-killed *Listeria monocytogenes* (HKLM; MOI 100; Invivogen, Toulouse, France), which activates the NF-κB/AP-1 pathway after binding to TLR2, as a positive control for the THP1-XBlue cell experiments, following the manufacturer’s protocol. Cells were incubated for 1 h with influenza virus A/PR/8/34 (H1N1) at an MOI of 2. To investigate exposure to *S. aureus* virulence factors, toxins and PAMPs were added to the cell suspension in RPMI 1640 medium at the following concentrations: PVL (0.05, 0.5, 2.5, and 5 µg/mL), protein A (0.1, 0.5, and 1 µg/mL), Hla (0.01, 0.1, and 1 µg/mL), HKSA (MOI 1, 10, 20, and 100), and PSMα1 and PSMα3 (1, 5, 10, and 25 µg/mL). The cells were then incubated for 24 h at 37 °C under 5% CO_2_.

Freshly isolated human monocytes were stimulated as described above. Human monocytes were used to test only the conditions that showed potentially significant effects in the continuous cell lines.

### 4.6. SEAP Reporter Assays

We collected the supernatant of THP1-XBlue cells and quantified SEAP using QuantiBlue reagent according to the manufacturer’s instructions (Invivogen, Toulouse, France). Absorbance at 650 nm was measured using an Asys UVM 340 microplate reader (Biochrom, Vindelle, France), and data were analyzed with DigiRead software (Biochrom, Vindelle, France). The optical density (OD) of the unexposed control group was subtracted from the OD of all samples.

### 4.7. Analysis of Cytokine Secretion

We collected the supernatants of human monocytes, and quantified human IL-1β, IL-6, and TNF-α using commercial ELISA kits according to the manufacturer’s protocols (Biosource, Nivelles, Belgium).

### 4.8. Flow Cytometry Analysis

To assess cytotoxicity, stimulated THP1 cells and human monocytes were stained with propidium iodide (PI; BD Biosciences, Le pont de Claix, France). Forward and side scatters (FSC and SSC, respectively) were used to exclude cells debris. Analyses were performed using a BD Accuri C6 (BD Biosciences, Le pont de Claix, France) and FlowJo Software (FlowJo, Ashland, OR, USA).

### 4.9. LDH Assay

To evaluate the cytotoxicity of influenza virus and *S. aureus* virulence factors on human monocytes, we measured the LDH release into supernatants using the Dimension vista 1500 (Siemens, Saint Denis, France).

### 4.10. Neutral Red Uptake by A549

A549 cells (ATCC^®^ CCL-185^TM^) were cultured and maintained at 37 °C and 5% CO_2_ in Dulbecco’s modified Eagle medium (DMEM), supplemented with 10% FBS and 100 U/mL pen-strep. To investigate the effects of monocyte supernatants on A549 cells, we plated A549 cells in 96-well tissue culture plates. Monocytes were stimulated as described above, and then pelleted. The supernatants were harvested and added to confluent epithelial cells. A549 cells were then incubated for 24 h at 37 °C and 5% CO_2_. The plates were then incubated for 45 min with medium containing 0.16% neutral red. Then the cells were washed, the dye was extracted using ethanol/citrate in each well, and the absorbance was read using an Asys UVM 340 (540 nm vs. 405 nm).

### 4.11. Statistics

Statistical analyses were performed using GraphPad Prism software (version 5.0, GraphPad Software, La Jolla, CA, USA). Data were analyzed by one-way ANOVA and Bonferonni post-hoc test. All values are expressed as mean (±SD). A *p* value of <0.05 was considered significant.
